# Simultaneous Nucleic Acids Detection and Elimination of Carryover Contamination With Nanoparticles-Based Biosensor- and Antarctic Thermal Sensitive Uracil-DNA-Glycosylase-Supplemented Polymerase Spiral Reaction

**DOI:** 10.3389/fbioe.2019.00401

**Published:** 2019-12-13

**Authors:** Yi Wang, Wei-wei Jiao, Yacui Wang, Lin Sun, Jie-qiong Li, Ze-ming Wang, Jing Xiao, Chen Shen, Fang Xu, Hui Qi, Yong-hong Wang, Ya-jie Guo, A-dong Shen

**Affiliations:** Key Laboratory of Major Diseases in Children, Ministry of Education, National Key Discipline of Pediatrics (Capital Medical University), National Clinical Research Center for Respiratory Diseases, Beijing Key Laboratory of Pediatric Respiratory Infection Disease, Beijing Pediatric Research Institute, Beijing Children's Hospital, Capital Medical University, National Center for Children's Health, Beijing, China

**Keywords:** isothermal nucleic acid amplification, polymerase spiral reaction, nanoparticle-based biosensor, *Klebsiella pneumoniae*, carryover contamination, LoD, rapid diagnosis, NB-ATSU-PSR

## Abstract

The current report devised a novel isothermal diagnostic assay, termed as nanoparticle-based biosensor (NB)- and antarctic thermal sensitive uracil-DNA-glycosylase (ATSU)-supplemented polymerase spiral reaction (PSR; NB-ATSU-PSR). The technique merges enzymatic digestion of carryover contaminants and isothermal nucleic acid amplification technique (PSR) for simultaneous detection of nucleic acid sequences and elimination of carryover contamination. In particular, nucleic acid amplification and elimination of carryover contamination are conducted in a single pot and, thus, the use of a closed-tube reaction can remove undesired results due to carryover contamination. For demonstration purpose, *Klebsiella pneumoniae* is employed as the model to demonstrate the usability of NB-ATSU-PSR assay. The assay's sensitivity, specificity, and practical feasibility were successfully evaluated using the pure cultures and sputum samples. The amplification products were detectable from as little as 100 fg of genomic DNAs and from ~550 colony-forming unit (CFU) in 1 ml of spiked sputum samples. All *K. pneumoniae* strains examined were positive for NB-ATSU-PSR detection, and all non-*K. pneumoniae* strains tested were negative for the NB-ATSU-PSR technique. The whole process, including rapid template preparation (20 min), PSR amplification (60 min), ATSU treatment (5 min), and result reporting (within 2 min), can be finished within 90 min. As a proof-of-concept methodology, NB-ATSU-PSR technique can be reconfigured to detect various target nucleic acid sequences by redesigning the PSR primer set.

## Introduction

Nucleic acid amplification is essential to most molecule testing strategies (Breaker, 2004). Polymerase chain reaction (PCR) is the most common methodology for nucleic acid amplification, but PCR-based techniques (such as conventional PCR, multiplex PCR, and real-time PCR) require complex experimental processes, sophisticated instruments, or trained personnel (Navarro et al., 2015). To solve these problems posed by classical PCR-based technologies, many isothermal amplification techniques [e.g., loop-mediated isothermal amplification (LAMP), multiple cross-displacement amplification (MCDA), cross-priming amplification (CPA), isothermal genome exponential amplification, and rolling circle amplification] have been reported, which rely on simpler apparatus, using only a single, moderate temperature (Wang et al., 2015; Zhao et al., 2015).

Although these established isothermal amplification technologies are able to offer extremely rapid results within a short time (30–90 min), they rely on complex design and/or special enzymatic activities to bypass the requirement for denaturing DNA strands; thus, their uptake is limited for common researchers (Law et al., 2015; Zhao et al., 2015). Moreover, current isothermal nucleic acid amplification assays require multiple primers at high concentrations; thus, they are prone to “mischief” (such as off-target hybrids, primer–dimers, and non-canonical folds) in the presence of polymerases and triphosphates. The consequences include low sensitivity, loss of signal, and poor reaction efficiency, and even as false negative and positive (Glushakova et al., 2015). As such, these isothermal amplification techniques involve rigorous optimization to obtain excellent performance.

Polymerase spiral reaction (PSR) is a novel nucleic acid sequence amplification-based technique, which can amplify target templates under the isothermal condition (60–67°C) with outstanding rapidity and high sensitivity and specificity (Liu et al., 2015). Unlike conventional isothermal amplification techniques, PSR does not rely on complex design and only requires one pair of primers (two primers) to achieve isothermal amplification. Thus, PSR assay effectively eliminates rigorous optimization and avoids these shortcomings yielding from traditional isothermal amplification assays (such as MCDA, LAMP, and CPA) that use multiple primers to achieve amplification. However, the result reporting of current PSR majorly relies on a colorimetric indicator, a tedious procedure, and complex optical apparatus (Gupta et al., 2017; Wang et al., 2019). Like other isothermal amplification techniques, result reporting using a colorimetric indicator is potentially subjective, gel electrophoresis analysis is a time-consuming process and easily generates cross-contamination, and real-time turbidity detection requires expensive instrument and often suffers from background interference (Sayad et al., 2016; Das et al., 2018). Herein, the current PSR form should be improved and simplified to widen its application in various fields.

The growing application of diagnostic methods has emphasized simplicity and speed as crucial criteria for adoption in molecular detection fields; thus, simplifying analysis tools of PSR technique is a major concern. In particular, nanoparticle-based biosensors [NBs; such as lateral flow biosensor (LFB)] have been increasingly used as alternative tools for analyzing various amplification products (such as PCR, LAMP, CPA, and MCDA amplicons) owing their simplicity, rapidness, and low cost (Gao et al., 2019; Li et al., 2019; Yao et al., 2019). Hence, the report firstly devised a novel PSR assay, which is assisted with NB, termed as NB-PSR. Moreover, further processing (such as reporting PSR result using gel electrophoresis or NB) requires the opening of reaction vessel, which carries high risk of carryover contamination. Thus, the new NB-PSR assay is further assisted with antarctic thermal-sensitive uracil-DNA-glycosylase (ATSU) for eliminating the carryover contamination and achieving reliable detection, termed as NB-ATSU-PSR assay.

Here, we expound the details on the NB-ATSU-PSR assay. Then, we validate the feasibility of the NB-ATSU-PSR assay by detecting *Klebsiella pneumoniae*, which is a notorious pathogen owing to a growth in the number of severe infections among the newborn, elderly, and immune-compromised people.

## Experimental Section

### Reagents and Materials

Isothermal amplification kit, visual detection reagent (VDR), and dUTP were obtained from Bei-Jing HaiTaiZhengYuan Co., Ltd. (Beijing, China). Anti-fluorescein isothiocyanate (FITC) and biotinylated bovine serum albumin (B-BSA) were obtained from Abcam Co., Ltd. (Shanghai, China). Dye (crimson red) streptavidin-coated polymer nanoparticles (DS-PNPs) were obtained from Bangs Laboratories, Inc. (Indiana, USA). ATSU was obtained from New England Biolabs, Inc. (Beijing, China). Biotin-14-dCTP and biotin-14-dATP were obtained from Thermo Scientific Co., Ltd. (Shanghai, China). The backing card, sample pad, conjugate pad, nitrocellulose (NC) membrane, and absorbent pad were obtained from the Jie-Yi Biotechnology Co., Ltd. (Shanghai, China).

### Preparation of Nanoparticle-Based Biosensor

**Figure 1:**
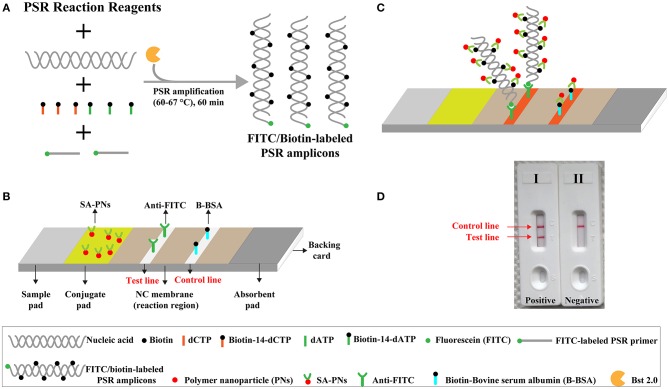
Outline of nanoparticle-based biosensor-supplemented polymerase spiral reaction assay (NB-PSR). **(A)** Outline of PSR with Ft* primer, biotin-14-dCTP, and biotin-14-dATP. **(B)** The detailed structure of NB. **(C)** The schematic illustration of the principle of NB for visualization of PSR products. **(D)** Interpretation of the results: (I) positive (two red bands, including test line and control line, appeared on the visual region of NB); (II) negative (only the control line region showed a red band).

NB includes a backing pad, an immersion pad, a conjugate pad, and an absorbent pad. DS-PNPs were gathered in the conjugate region (Figure 1). Anti-FITC and B-BSA were deposited onto test line (TL) and control line (CL), respectively. A 1 μl of aliquot of reaction products is deposited to the sample application region of the biosensor, and a 60 μl of aliquot of running buffer (10 mM of PBS, pH 7.4 with 1% Tween 20) was also deposited to the same region of biosensor. Then, the biosensor is able to absorb the whole running buffer. Thus, the product detection is indicated by the presence of crimson red lines on the NC regions.

### Primer Design

*Klebsiella pneumoniae* was used as the model target for validating the feasibility of NB-ATSU-PSR technology. According to the design principle of a PSR primer, two primers, including Ft and Bt, were designed according to the *rscA* gene of the *K. pneumoniae* (Figure S1). In the NB-PSR system, only a PSR primer (Ft or Bt), which is involved in PSR amplification, is labeled with hapten (such as fluorescein and FITC) at the 5′ end. The new Ft or Bt primer is termed as Ft^*^ or Bt^*^ (Figure S1B). In this report, the Ft^*^ primer is employed as the model primer to validate the availability of NB-PSR methodology. All of the oligomers were synthesized and purified by TsingKe Biotech Co., Ltd. (Beijing, China) at high-performance liquid chromatography (HPLC) purification grade.

### Polymerase Spiral Reaction and Antarctic Thermal Sensitive Uracil-DNA-Glycosylase-Supplemented Polymerase Spiral Reaction

PSR amplifications were performed in 25 μl of mixture containing 12.5 μl of 2× of the supplied buffer, 3.2 μM each of Ft^*^ and Bt, 1.4 mM of dATP, 1.4 mM of dCTP, 1.4 mM of dGTP, 1.4 mM of dTTP, 0.15 μl of biotin-14-dCTP (50 mM), 0.15 μl of biotin-14-dATP (50 mM), 1 μl (8 U) of *Bst 2.0* DNA polymerase, and 1 μl of DNA template.

ATSU-PSR amplifications also were performed in 25 μl of mixture containing 12.5 μl of 2× of the supplied buffer, 3.2 μM each of Ft^*^ and Bt, 1.4 mM of dATP, 1.4 mM of dCTP, 1.4 mM of dGTP, 0.7 mM of dTTP, 0.7 mM of dUTP, 0.15 μl of biotin-14-dCTP (50 mM), 0.15 μl of biotin-14-dATP (50 mM), 0.3 μl (0.3 U) of ATSU, 1 μl (8 U) of *Bst 2.0* DNA polymerase, and 1 μl of DNA template.

In particular, a total of three monitoring techniques, including VDR, real-time turbidity (LA-320), and NB, were employed for confirming and demonstrating the amplification of PSR-based assays. In addition, PSR temperatures ranging from 60 to 67°C at 1°C interval were tested for confirming the optimal PSR. PSR amplification mixtures with 1 μl of genomic template of *Listeria monocytogenes* and *Shigella flexneri* were employed as negative controls (NC). PSR mixtures with 1 μl of double distilled water (DW) were used as a blank control (BC).

### Sensitivity of Polymerase Spiral Reaction Assays

To test assay's sensitivity, analytical sensitivity of PSR methods was examined using serial dilutions (10 ng, 1 ng, 100 pg, 10 pg, 1 pg, 100 fg, and 10 fg per microliter) of purified genomic templates of *K. pneumoniae* reference strain [American Type Culture Collection (ATCC) BAA-2164], and 1 μl of aliquot of each dilution was added to the PSR mixtures. The analytical sensitivity was confirmed as the last dilution of each positive test, and each dilution was examined in triplicate.

### Simulating Carryover Contamination

PSR amplification products, which were obtained from 1 pg μl^−1^ without ATSU, were employed as the templates for simulating carryover contamination. Firstly, the PSR amplification products were quantitated using ultraviolet spectrophotometer (NanoDrop ND-1000, Caliber, Beijing, China). Secondly, the quantitated PSR products were serially diluted (10-fold) arranging from 1 × 10^−13^ to 1 × 10^−20^ g μl^−1^. One microliter of aliquot of artificially contaminated product was added to ATSU-PSR amplification mixtures.

### Prevention of Carryover Contamination by Antarctic Thermal-Sensitive Uracil-DNA-Glycosylase-Supplemented Polymerase Spiral Reaction

To validate the capability of ATSU-PSR method to eliminate the unwanted results owing to carryover contaminants in detecting target templates, we compared ATSU-PSR technique with normal PSR methods (ATSU-PSR without ATSU enzyme digestion) by adding 1 μl of simulated carryover contamination of 1 × 10^−18^ g μl^−1^ and 1 μl of diluted templates in the same tube. Here, total mass (1 × 10^−18^ g) of simulated carryover contaminants was equal to a 0.2-μm-diameter aerosol droplet, which was not effectively prevented by either high-efficiency particulate air filters in the biosafety cabinets or fibrous pipette tip filters (Le Rouzic, 2006; Barhate and Ramakrishna, 2007). Hence, the total mass (1 × 10^−18^ g) of contaminants was employed as the source of simulating carryover contaminants for ATSU-PSR and normal PSR amplification. Analytical sensitivity of PSR in the absence of ATSU digestion and in the presence of ATSU digestion before amplification was compared to verify whether the ATSU-PSR method developed here was capable of effectively removing undesired results.

### Specificity of Antarctic Thermal-Sensitive Uracil-DNA-Glycosylase-Supplemented Polymerase Spiral Reaction Assay

To determine the analytical specificity of NB-ATSU-PSR for *K. pneumoniae* detection, we perform the NB-ATSU-PSR method on at least 5 ng of DNAs, which were extracted from a panel of *K. pneumoniae* and non-*K. pneumoniae* bacteria (Table 1). In particular, we used *K. pneumoniae* reference strain ATCC BAA-2164 as a positive strain and DW as the BC.

**Table 1:** Bacterial strains used in this study.

**Bacteria**	**Strain no. (source of strains)^a^**	**No. of strains**	**NB-ATSU-PSR result^b^**
*Klebsiella pneumoniae*	ATCC BAA-2146	1	P
	ATCC BAA-1705	1	P
	Isolated strains	17	P
*Non-K. pneumoniae*			
*Streptococcus pneumoniae*	ATCC 700674	1	N
*Enterococcus faecalis*	ATCC 35667	1	N
*Enterococcus faecium*	Isolated strain	1	N
*Plesiomonas shigelloides*	Isolated strain	1	N
*Staphylococcus epidermidis*	Isolated strain	1	N
*Staphylococcus aureus*	Isolated strain	1	N
*Pseudomonas aeruginosa*	Isolated strain	1	N
*Shigella dysenteriae*	Isolated strain	1	N
*Shigella boydii*	Isolated strain	1	N
*Shigella flexneri*	Isolated strain	1	N
*Shigella sonnei*	Isolated strain	1	N
*Aeromonas hydrophila*	Isolated strain	1	N
*Enterobacter cloacae*	Isolated strain	1	N
*Bntorobater sakazakii*	Isolated strain	1	N
*Campylobacter jejuni*	ATCC 33291	1	N
*Enterotoxigenic Escherichia coli*	Isolated strains	1	N
*Listeria monocytogenes*	ATCC EGD-e	1	N
*Listeria innocua*	Isolated strain	1	N
*Vibrio parahaemolyticus*	Isolated strain	1	N
*Acinetobacter baumannii*	Isolated strain	1	N
*Neisseria meningitidis*	Isolated strain	1	N
*Bordetella pertussis*	Isolated strain	1	N
*Stenotrophomonas maltophilia*	Isolated strain	1	N

a *ATCC, American Type Culture Collection*.

b *P, positive; N, negative. Only templates extracted from K. pneumoniae strains were detected by the nanoparticle-based biosensor–antarctic thermal-sensitive uracil-DNA-glycosylase-supplemented polymerase spiral reaction (NB-ATSU-PSR assay), indicating the extremely high selectivity of the assay*.

### Evaluation of the Feasibility of Nanoparticle-Based Biosensor–Antarctic Thermal-Sensitive Uracil-DNA-Glycosylase-Supplemented Polymerase Spiral Reaction Technique

In this report, we tested the lowest number of bacterial cells detectable in spiked samples by the NB-ATSU-PSR technique. Firstly, we prepared the serial 10-fold dilutions of reference strain cells, which were used for spiking sputum samples. Then, the artificially seeded sputum samples were prepared using five *K. pneumoniae*-negative sputa, and these clinical specimens were obtained from five different patients with patients' informed consent (Clinical Laboratory of Shougang Hospital, Peking University).

Briefly, an equal volume of 0.1% dithiothreitol (Sigma) was added into five sputum samples and then vortexed (10 min) and incubated (10 min) at room temperature (~25°C). The homogenate (artificially contaminated sputa) was divided into 0.9 ml, and 0.1 ml of aliquots of appropriate *K. pneumoniae* suspension (reference strain ATCC-BAA-2164) was placed into the spiked samples. Thus, the spiked sputum samples were adapted to 0.011–11,000 reference strain cells per milliliter; 0.1 ml of aliquots of artificially contaminated sputa was subjected to extract the genomic DNAs, and a volume of 4 μl of extracted DNA was used as templates for NB-ATSU-PSRs.

## Results

### Development of Nanoparticle-Based Biosensor-Assisted Polymerase Spiral Reaction Assay

The NB-PSR design and reaction mechanism are depicted in Figure 1. Here, the Ft^*^ primer is employed as the model primer to validate the availability of NB-PSR methodology, which is labeled with hapten (such as fluorescein and FITC) at the 5′ end. In addition, two components, including biotin-14-dCTP and biotin-14-dATP, were added into the PSR amplification mixtures.

During the amplification stage, the Ft^*^ primer, which is a hapten-labeled primer, anneals to the target genomic template and is extended by isothermal amplification enzyme (*Bst* 2.0 polymerase). Thus, biotin-14-dCTP and biotin-14-dATP are successfully incorporated into the PSR amplicons. As a result, a large number of double-labeled detectable products, which are simultaneously labeled with hapten (FITC) and biotin, are therefore produced (Figure 1A). These double-labeled detectable products derive from a hapten-labeled primer, biotin-14-dCTP, and biotin-14-dATP. In particular, the more details of conventional PSR process have been thoroughly displayed in a previous report (Liu et al., 2015).

The principle of NB visualization of target PSR products is exhibited in Figures 1B,C. Here, PSR amplicons is FITC labeled and biotin labeled, as derived from the FITC-labeled primer, biotin-labeled dCTP, and biotin-labeled dATP. The PSR mixtures (1 μl aliquots) are loaded into the sample region of the biosensor, and then 60 μl of aliquots of diluent buffer also is deposited on the same area. The diluent buffer moves along the NB by capillary action and rehydrates the immobilized detector reagents (DS-PNPs) at the conjugate sample. The NB detects the double-labeled PSR products through recognition of the FITC tag at the end of the PSR amplicons, and FITC tag is specially captured by anti-FITC body at the TL. Then, detector reagent (DS-PNPs) accumulates on the TL through streptavidin/biotin interaction (Figures 1B,C). Thus, a visual crimson red line is produced at the test area of biosensor. Particularly, excess detector reagents are captured by fixed biotin-BSA (B-BSA) at the control area, which confirms the proper function of biosensor (Figure 1C).

The interpretation of detection result is based on the appearance of crimson red bands on the biosensor (Figure 1D). The presence of two crimson red lines, including TL and CL, indicates a positive result for target detection (Figure 1D, biosensor 1). Only a crimson red band (CL) appears on the biosensor, reporting a negative result (Figure 1D, biosensor 2). The colorimetric signal is seen by naked eyes within 2 min (Figure 1D). In all the analyses conducted, CL must appear in the chromatography control region, because the proper functioning of biosensor is verified by the CL formation.

### Confirmation of Polymerase Spiral Reaction Products

To validate the feasibility of a PSR primer set designed in this study, we conducted the conventional PSRs under the isothermal condition (63°C) in the presence of or absence of templates for 1 h. By using the biosensor, TL and CL simultaneously appear on the detection regions of the biosensor, and only CL appears on the biosensor for blank and negative controls (Figure S2A). By using VDR (colorimetric indicator), the color of the PSR solution changed from colorless to light green in the positive PSR, and the PSR solution remained colorless in the negative PSR (Figure S2B). These results demonstrated that the PSR primer set designed in this report can successfully amplify the target templates and thus is an available candidate for establishing the NB-ATSU-PSR technique.

### The Optimization of Reaction Temperature Polymerase Spiral Reaction Primer Set

The conventional standardization of the PSR was carried out to compare the rate of amplification reaction. The test was performed at different temperatures (60–67°C, with 1°C interval) for 60 min with 1 pg of *K. pneumoniae* (ATCC BAA-2164) templates. The amplification of target sequences occurred at all examined temperatures (Figure S3). The optimum temperature recorded for PSR assay was 63°C, because the faster amplification of PSR reaction was observed (Figure S3). Thus, the assay temperature of 63°C was used for all examinations in this study.

### Sensitivity of Nanoparticle-Based Biosensor–Polymerase Spiral Reaction Method in Pure Cultures

**Figure 2:**
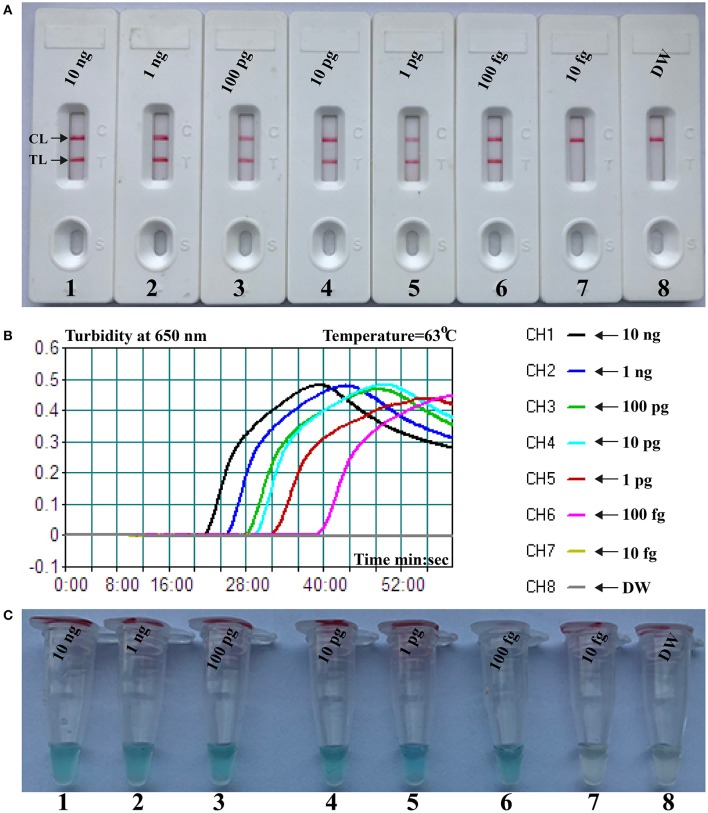
Sensitivity of nanoparticle-based biosensor-supplemented polymerase spiral reaction (NB-PSR) assay using serially diluted *Klebsiella pneumoniae* [American Type Culture Collection (ATCC) BAA-2146] templates. Biosensor **(A)** Real-time turbidity **(B)** and colorimetric indicator **(C)** were applied for analyzing the PSR products. Biosensor/signal/tube 1, 2, 3, 4, 5, 6, and 7 represent the genomic DNA levels of 10 ng μl^−1^, 1 ng μl^−1^, 100 pg μl^−1^, 10 pg μl^−1^, 1 g μl^−1^, 100 fg μl^−1^, and 10 fg μl^−1^, respectively. Biosensor/signal/tube 8, blank control (DW).

The limit of detection (LoD) of NB-PSR assay was confirmed using equivalent quantities of templates extracted from pure cultures (*K. pneumoniae* strain ATCC BAA-2164) with different dilutions. As shown in Figure 2, sensitivity of NB-PSR for *K. pneumoniae* detection was 100 fg of genomic templates per vessel (Figure 2A). With the use of the PSR by self-trail, LoD of PSR was further demonstrated by real-time turbidity analysis (Figure 2B) and by visual inspection of reaction mixtures with VDR (Figure 2C). The sensitivity of PSR method was also 100 fg of template per reaction using the real-time turbidity and VDR analysis (Figures 2B,C), which was in complete accordance with NB detection (Figure 2A).

### Antarctic Thermal Sensitive Uracil-DNA-Glycosylase-Supplemented Polymerase Spiral Reaction Assay

**Figure 3:**
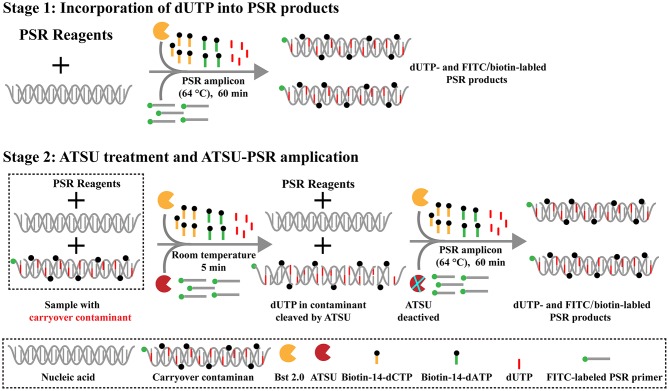
Schematic illustration of the principle of antarctic thermal-sensitive uracil-DNA-glycosylase-supplemented polymerase spiral reaction (ATSU-PSR) technique for eliminating carryover contamination. Two steps are needed for ATSU-PSR technique for removing carryover contamination. During the first stage, all PSR complicons labeled with dUTP in the presence of *Bst 2.0* enzyme and dUTP. During the second stage, all subsequent ATSU-PSR amplifications are digested using ATSU, which specifically cleave carryover contaminants by removing uracil in amplicons from previous reactions. Importantly, ATSU is heat inactivated during the PSR amplification stage (63°C), and the digested contaminants are degraded, ensuring that only the target templates are amplified. In addition, three components, including fluorescein isothiocyanate (FITC)-labeled primer, biotin-14-dCTP, and biotin-14-dATP, are added into ATSU-PSR mixtures for forming the biotin- and FITC-attached duplex products.

Here, we firstly reported the integration of ATSU digestion with PSR (STSU-PSR) in a one-pot, closed-vessel reaction to remove the unwanted amplifications generated from carryover contaminants (Figure 3). ATSU-PSR assay eliminates the carryover contaminants at two stages and requires two additional components (ATSU enzyme and dUTP) compared with conventional PSR technique. At the first stage, dUTP is added into all PSR amplification mixtures; thus, uracil is incorporated into all PSR products. At the second stage, we performed the ATSU treatment and the PSR in a one-pot process. Prior to isothermal amplification, the PSR mixture is digested by a heat-labile ATSU enzyme for 5 min at room temperature. Thus, ATSU selectively cleaves uracil bases from any contaminating amplicons, which leaves behind abasic sites. These abasic sites are able to cause rapid degradation of carryover templates via hydrolysis at the phosphate backbone when the temperature is higher than 50°C. In the PSR (60–67°C), the resulting abasic sites are able to block replication by Bst DNA polymerases, effectively preventing the contaminating amplicons from re-amplification. Importantly, the uracil-free target genomic template (i.e., the natural DNA) cannot be digested by ATSU enzyme, and thus, it remains completely unaffected. Moreover, three components (FITC-labeled Ft primer [Ft^*^], biotin-14-dCTP, and biotin-14-dATP) are placed into the PSR amplification mixtures to form the FITC-/biotin-labeled amplicons, which can be visually indicated using NB devised in this report (Figures 1, 3).

### Antarctic Thermal Sensitive Uracil-DNA-Glycosylase-Supplemented Polymerase Spiral Reaction Method Detects Simulated Carryover Contaminants

**Figure 4:**
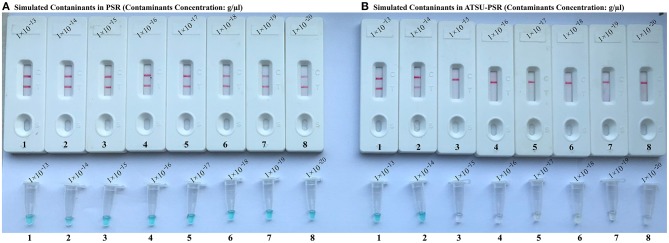
Control of carryover contamination in uracil-DNA-glycosylase-supplemented polymerase spiral reaction (ATSU-PSR) assay. Sensitivity of PSR **(A)** and ATSU-PSR **(B)** using serial dilution of simulated carryover contamination (dUTP-incorporated PSR amplicons, concentration diluted from 1 × 10^−13^, 1 × 10^−14^, 1 × 10^−15^, 1 × 10^−16^, 1 × 10^−17^, 1 × 10^−18^, 1 × 10^−19^, and 1 × 10^−20^ g μl^−1^) as determined using biosensor (top row) and visual detection reagent (bottom row).

To confirm that PSR products (dUTP-incorporated PSR amplicons) from previous reactions are able to contaminate new PSR amplifications and cause the false-positive results. In this report, the conventional PSR and the new ATSU-PSR methods were conducted using serial diluted PSR products (simulated carryover contaminants) with concentrations ranging from 1 × 10^−13^ to 1 × 10^−20^ g μl^−1^. The conventional PSR assay, which in the absence of ATSU digestion, was able to detect as little as 1 × 10^−20^ g of simulated carryover contaminants per reaction. In contrast, the new ATSU-PSR assay, in the presence of ATSU digestion, only detected 1 × 10^−14^ g of simulated carryover contaminants per reaction. These results confirm that the new ATSU-PSR assay can eliminate the PSR amplification up to 10^6^-fold higher concentrations of contaminants (1 × 10^−20^ g) than do the conventional PSR assay (Figure 4). Particularly, low abundance of PSR contaminants (1 × 10^−18^ g of 0.2-μm-diameter aerosol droplet), which cannot be removed by fibrous pipette tip filters and can yield unwanted positive amplifications, is successfully prevented by the new ATSU-PSR assay. Herein, our results demonstrated that the new ATSU-PSR assay was capable of significantly reducing the likelihood of false-positive amplification generating from carryover contaminants.

### Antarctic Thermal Sensitive Uracil-DNA-Glycosylase-Supplemented Polymerase Spiral Reaction Method Eliminates False-Positive Results

**Figure 5:**
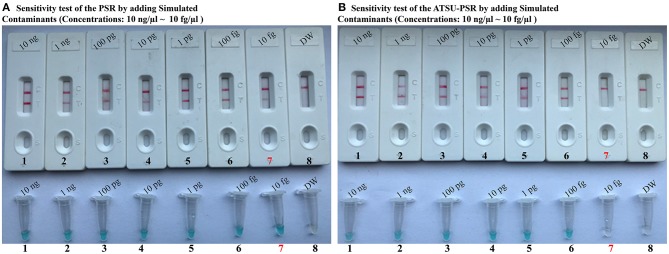
Uracil-DNA-glycosylase-supplemented polymerase spiral reaction (ATSU-PSR) assay prevents false-positive result due to carryover contaminants. Sensitivity of conventional PSR **(A)** and ATSU-PSR technique **(B)** using serial dilutions (10 ng μl^−1^, 1 ng μl^−1^, 100 pg μl^−1^, 10 pg μl^−1^, 1 g μl^−1^, 100 fg μl^−1^, and 10 fg μl^−1^) of American Type Culture Collection (ATCC) BAA-2146 and 1 × 10^−18^ g μl^−1^ of simulated carryover contamination (dUTP-incorporated PSR products) as determined using biosensor and visual detection reagent.

To further confirm that the new ATSU-PSR assay can reduce the likelihood of unwanted amplifications due to the carryover contaminates, we examined the sensitivity of ATSU-PSR and PSR assays using serial dilution of the reference *K. pneumoniae* (ATCC BAA-2164); simultaneously, the stimulated carryover contaminants at the level of 1 × 10^−18^ g also were placed into PSR mixtures. As shown in Figure 5, all samples displayed positive amplification in the PSR method in the absence of ATSU digestion, even samples containing undetectable genomic templates (<100 fg per tube), which were regarded as false-positive results (Figure 5A). In contrast, the ATSU-PSR assay's sensitivity was in complete accordance with the aforementioned LoD evaluation (Figures 2, 5B). These results further confirmed that ATSU-PSR technique devised in the report could effectively prevent undesired results yielding from carryover contamination, and traditional PSR assay lacked the capability of eliminating carryover contamination and thus did not correctly verify the assay's sensitivity.

### Specificity of Nanoparticle-Based Biosensor–Antarctic Thermal-Sensitive Uracil-DNA-Glycosylase-Supplemented Polymerase Spiral Reaction Assay

**Figure 6:**
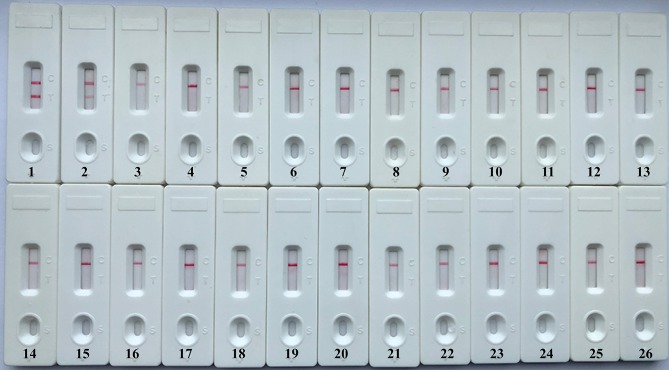
Specificity of uracil-DNA-glycosylase-supplemented polymerase spiral reaction (ATSU-PSR) for identifying *Klebsiella pneumoniae*. Biosensor 1, *K. pneumoniae* (ATCC BAA-2146). Biosensor 2, *K. pneumoniae* (isolate strain). Biosensors 3–25, *Streptococcus pneumoniae*; *Enterococcus faecalis*; *Enterococcus faecium*; *Plesiomonas shigelloides*; *Staphylococcus epidermidis*; *Staphylococcus aureus*; *Pseudomonas aeruginosa*; *Shigella dysenteriae*; *Shigella boydii*; *Shigella flexneri*; *Shigella sonnei*; *Aeromonas hydrophila*; *Enterobacter cloacae*; *Bntorobater sakazakii*; *Campylobacter jejuni*; *Enterotoxigenic Escherichia coli*; *Listeria monocytogenes*; *Listeria innocua*; *Vibrio parahaemolyticus*; *Acinetobacter baumannii*; *Neisseria meningitidis*; *Bordetella pertussis*; and *Stenotrophomonas maltophilia*. Biosensor 26, double distilled water (DW).

A specificity test was performed by using different strains, including 19 *K. pneumoniae* strains and 23 non-*K. pneumoniae* strains (i.e., *Streptococcus pneumoniae, Staphylococcus aureus, L. monocytogenes*, and *Salmonella*). As shown in Figure 6 and Table 1, all positive results were obtained only from *K. pneumoniae* strains tested, whereas the non-*K. pneumoniae* isolates yielded negative results. No cross-reactions were observed in the NB-ATSU-LFB assay.

### Nanoparticle-Based Biosensor–Antarctic Thermal-Sensitive Uracil-DNA-Glycosylase-Supplemented Polymerase Spiral Reaction Method to *Klebsiella pneumoniae* in Spiking Sputum Samples

To verify the applicability of NB-ATSU-PSR method as a valuable diagnostic tool, we evaluated the NB-ATSU-PSR assay using the spiking sputum samples with *K. pneumoniae*. As shown in Figure S4, NB-ATSU-PSR method yielded the positive results when the contaminated numbers of *K. pneumoniae* reference strain (ATCC BAA-2164) were more than 550 colony-forming unit (CFU) ml^−1^ (~more than 11 CFU/reaction). The negative control (non-contaminated sputum samples) produced negative results.

## Discussion

PSR, which was is novel isothermal amplification methodology, was devised for offering simplified formats of nucleic acids detection (Liu et al., 2015). Particularly, indicating PSR results is a pivotal concern as simplifying detection tools is a major concern. Traditional monitoring techniques, including agarose gel electrophoresis, turbidimetry and visual detection, are not specific to target PSR amplicons; thus, they did not differentiate specific amplification from non-specific amplification (Wozniakowski et al., 2017). Moreover, these detection methods used for PSR assay required an additional analysis step (gel electrophoresis), optical instrument (i.e., turbidimeters), and special visual reagents (such as fluorescent), which restrict its wider application in various fields, such as point-of-care testing, “on-site” detection, and field diagnosis.

Recently, nanoparticle-based LFB (NB) has been developed and employed as an alternative detection tool for analyzing various amplification results, such as PCR and LAMP (Chua et al., 2011; Wang et al., 2017). As a superior analysis tool, the NB overcomes these shortcomings posed by conventional detection technique, owing to the important advantages of being user-friendly, pollution free, visual result interpretation, and rapid signal output in poor-resource settings. Here, we firstly reported a novel PSR-based assay, termed as NB-assisted PSR technique (NB-PSR). In the NB-PSR assay (Figure 1), only a core primer (Ft or Bt) involved in isothermal reaction is labeled with hapten (FITC) at the 5′ end. Two additional components, including biotin-14-dCTP and biotin-14-dATP, are added into the PSR amplification mixtures. During the amplification stage, the PSR amplicons are simultaneously labeled with FITC and biotin. The end labeled with FITC is specially captured by the anti-FITC antibody, which is immobilized at the TL of the biosensor. The biotin tags, which are incorporated into PSR amplicons labeled using biotin-14-dCTP and biotin-14-dATP, are capable of binding the streptavidin-conjugated color nanoparticles for visualization. The PSR amplification results are indicated as a colorimetric band visible by the unaided eye within 2 min (Figure 1D).

To achieve the analysis of PSR products using NB, the opening of reaction vessel is an essential step. The aerosol droplets of different sizes, which include high concentration of PSR products, are produced. Owing to its high sensitivity, the PSR assay is particularly vulnerable to carryover contamination. As such, the carryover contamination is one of the most important concerns affecting PSR method because it can cause false-positive results. Therefore, when the NB-PSR technique is used for detection of various target sequences, the possibility of carryover contamination should be removed. In this report, our data suggested that a trace amount of carryover contaminants (1 × 10^−18^ g/reaction) generated from previous PSRs is able to cause unwanted results (Figure 4). In order to achieve the accurate and reliable NB-PSR diagnostic methodology, elimination of carryover contamination is a vital issue.

Here, this study firstly reported the elimination of carryover contamination in PSR-based methodologies using ATSU enzyme and dUTP (Figure 3). At the first stage, the component (dUTP) is incorporated instead of dTTP into all PSR amplicons in the ATSU-PSR system. Importantly, PSR amplification and ATSU treatment are performed in a one-pot step. Prior to PSR, PSR mixtures are digested at room temperature for 5 min; thus, the ATSU enzyme can specifically digest uracil bases from carryover contaminants. The abasic sites cause degradation of contaminants via hydrolysis at the phosphate backbone, which successfully prevents the PSR replication by *Bst* 2.o polymerase during the reaction stage, thus hindering carryover contaminants from re-amplification (Figure 3). Importantly, natural target templates are uracil-free nucleic acids; thus, they remain completely unaffected. In the NB-ATSU-PSR system, ATSU is a heat-labile enzyme and is used for catalyzing the removal of uracil based from uracil-containing contaminants. In particular, ATSU enzyme automatically and rapidly deactivates when the PSR amplification is performed at an elevated temperature (i.e., >60°C). Hence, the application of ATSU enzyme enables the ATSU-PSR method to be conducted in a single closed tube. The genuine PSR products subsequently yielded from the target templates are not affected during the isothermal amplification stage, thus permitting PSR amplification to normally proceed. The whole process, including rapid template preparation (20 min), PSR amplification (60 min), ATSU treatment (5 min), and result reporting (within 2 min), can be finished within 90 min.

As a proof-of-concept, the feasibility of NB-ATSU-PSR technique was successfully demonstrated by detecting *K. pneumoniae* templates in pure cultures and spiked sputum samples. The organism is an important hospital-acquired bacterium and causes morbidity and mortality among immunocompromised patients, newborn infants, and older adults. Herein, it is important to rapidly detect this pathogen in a clinical laboratory to enable prevention and to control the spread of the *K. pneumoniae* infections. In this report, a set of two PSR primers (Ft and Bt primers) was designed for detection of the target bacterium according to the specific gene **rcsA** (Figure S1). Then, the usability of a PSR primer set was evaluated by performing the PSRs in the absence or presence of *K. pneumoniae* templates under the isothermal conditions (Figure S2). A total of eight reaction temperatures were tested (60–67°C, 1°C interval) for selecting the optimal PSR amplification temperature, because the reaction temperature was extremely important factor for isothermal detection assay (Figure S3). Our results demonstrated that the faster reaction was obtained at the assay temperature of 63°C, which thus was used as the optimal amplification temperature for the rest of PSR-based reactions conducted in this report. The assay's sensitivity was 100 fg per reaction when NB-ATSU-PSR detected *K. pneumoniae* in pure cultures (Figures 2, 5). Although the assay's sensitivity obtained using VDR and real-time turbidity was in complete accordance with NB, the biosensor is likely the preferred monitoring method, as reporting the detection results using biosensor does not require any special instruments or reagents and is less subjective. All *K. pneumoniae* strains examined in this study were correctly identified by NB-ATSU-PSR assay, and no positive results were observed in the assay of non-target strains, suggesting the high selectivity of NB-ATSU-PSR (Figure 6). In addition, the practical application of NB-ATSU-PSR technique was also successfully evaluated by detecting *K. pneumoniae* in sputum samples with high sensitivity and specificity (Figure S4).

## Conclusion

In this report, we devised a novel PSR assay, termed as NB- and ATSU-supplemented PSR. NB-ATSU-PSR technique merges enzymatic digestion of carryover contaminants and isothermal nucleic acid amplification technique (PSR) for simultaneous detection of nucleic acid sequences and elimination of carryover contamination. For demonstration purpose, *K. pneumoniae* is employed as the model to demonstrate the usability of NB-ATSU-PSR assay. The assay's sensitivity, specificity, and practical feasibility were successfully evaluated using the pure cultures and sputum samples. As a proof-of-concept methodology, the NB-ATSU-PSR technique can be reconfigured to detect various target nucleic acid sequences by redesigning the PSR primer set.

## Data Availability Statement

The raw data supporting the conclusions of this article will be made available by the authors, without undue reservation, to any qualified researcher.

## Author Contributions

YiW conceived and designed the experiments and performed the software. YiW, YaW, LS, JL, ZW, WJ, JX, CS, FX, HQ, YoW, YG, and AS performed the experiments, contributed the reagents, materials, and analysis tools. YiW, YaW, and WJ analyzed the data. YiW and AS wrote the paper.

### Conflict of Interest

The authors declare that the research was conducted in the absence of any commercial or financial relationships that could be construed as a potential conflict of interest.
