# Supplementary material for: Simultaneous Nucleic Acids Detection and Elimination of Carryover Contamination With Nanoparticles-Based Biosensor- and Antarctic Thermal Sensitive Uracil-DNA-Glycosylase-Supplemented Polymerase Spiral Reaction
Source: Front Bioeng Biotechnol. 2019 Dec 13;7:401. doi: 10.3389/fbioe.2019.00401 (PMC6923221; doi:10.3389/fbioe.2019.00401)
Supplement: Supplementary file 1 [file Data_Sheet_1.pdf]

Supplementary Material

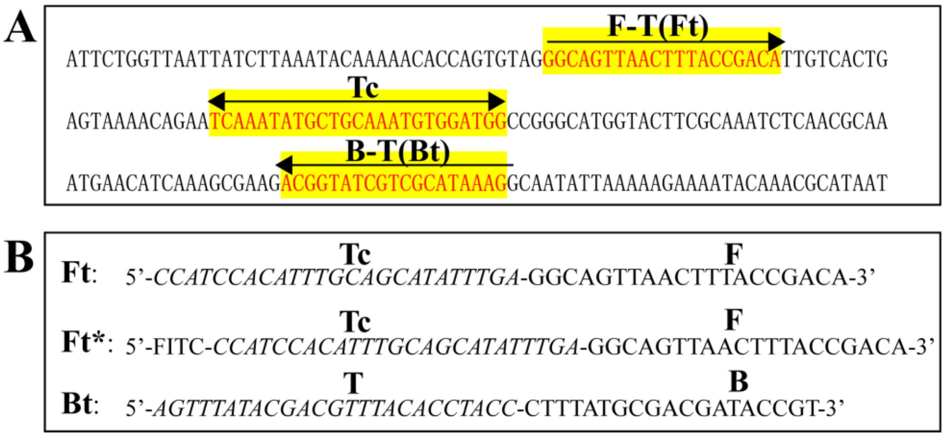

Figure S1. **Primer design for the PSR assay.** (**A**), Nucleotide sequence of the ***rscA*** gene (**part**) and locations of the primers are underlined. (**B**), Primer sequences targeting ***rscA***.

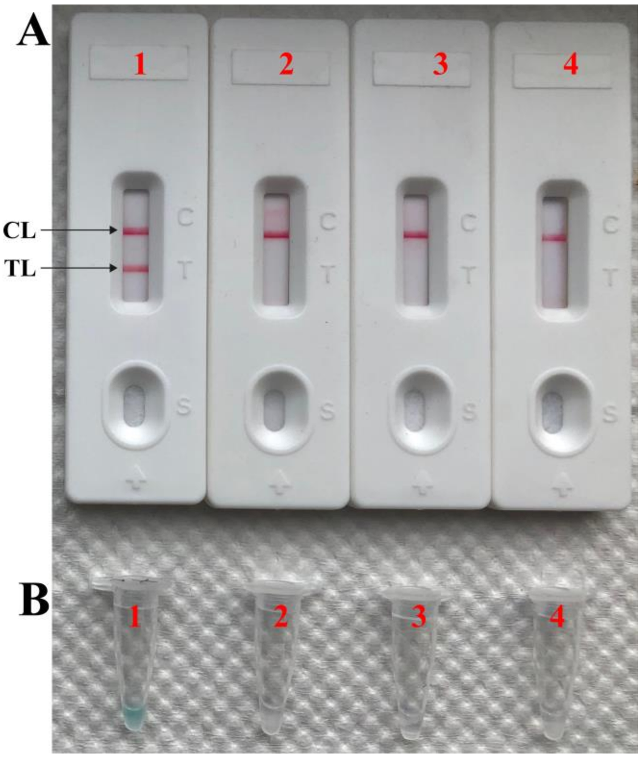

Figure S2. Detection and confirmation of PSR products. (**A**), The biosensor applied for visual detection of PSR products. **(B)**, Colorimetric indicator (VDR) applied for visual determination of PSR amplification products. Biosensor/Tube 1, positive amplification of PSR method (*K. pneumoniae*, ATCC BAA-2146); Biosensor/Tube 2, negative amplification of PSR method (*L. monocytogenes*); Biosensor/Tube 3, negative amplification of PSR assay (*S. flexneri*); Biosensor/Tube 4, blank control (DW).

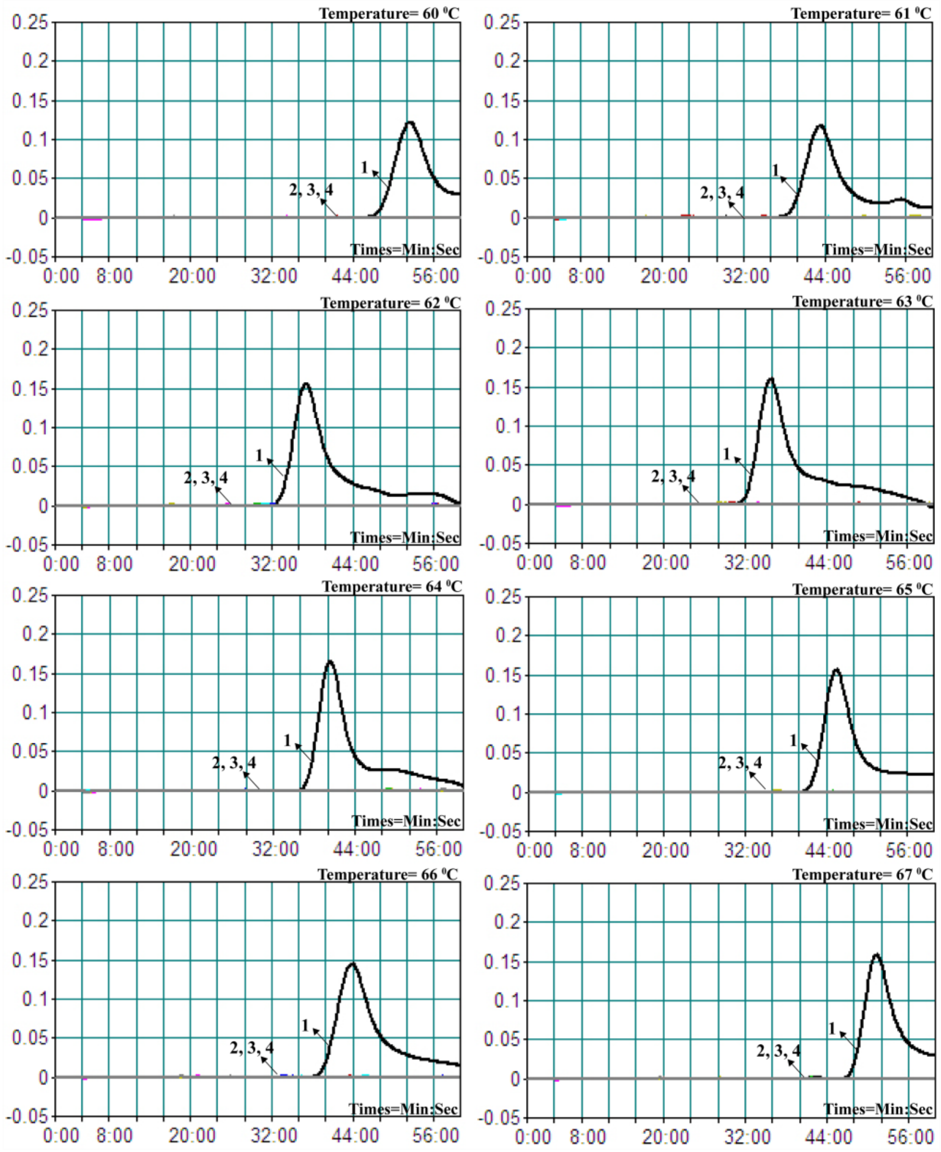

Figure S3. Optimal reaction temperature for PSR primer set. PSR reactions were monitored by real-time measurement of turbidity (LA-320c), and the threshold value was 0.1. The turbidity of >0.1 was regarded as positive amplification. Eight kinetic graphs were observed at different temperatures (60°C-67°C, 1°C intervals) with target templates at the level of 1 pg per tube. Signal 1, positive amplification of PSR assay (*K. pneumoniae*, ATCC BAA-2146); Signal 2, negative amplification of PSR assay (*L. monocytogenes*); Signal 3, negative amplification of PSR assay (*S. flexneri*); Signal 4, blank control (DW).

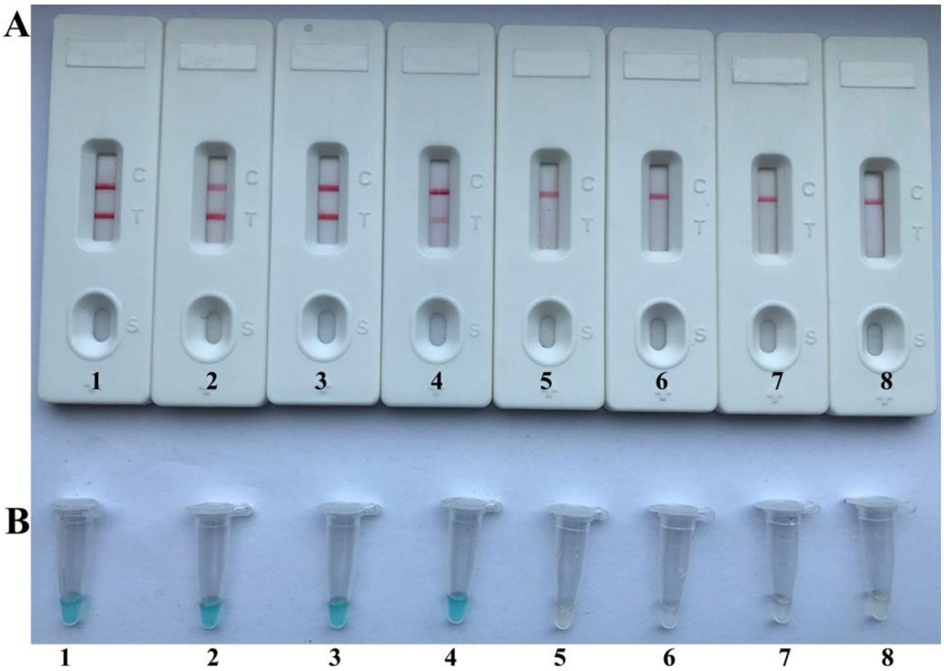

Figure S4. Sensitivity of ATSU-PSR for detecting *K. pneumoniae* in sputum samples.

Biosensor (A)/Tubes (B) 1-8 represented the cell levels of 11000 CFU per reaction (~ 550000 CFU/mL), 1100 CFU per reaction (~ 55000 CFU/mL), 110 CFU per reaction (~ 5500 CFU/mL), 11 CFU per reaction (~ 550 CFU/mL), 1.1 CFU per reaction (~ 55 CFU/mL), 0.11 CFU per reaction (~ 5.5 CFU/mL), 0.011 CFU per reaction (~ 0.55 CFU/mL), negative control (non-contaminated sputum samples). The cell levels of 11000 CFU, 1100 CFU, 110 CFU and 11 CFU per reaction produced the positive amplifications.
